# Transthoracic management of liver dome hydatid cyst: a single-center experience in a North African country

**DOI:** 10.3389/fsurg.2025.1689825

**Published:** 2025-11-21

**Authors:** Laila Jedidi, Senda Ben Lahouel, Yosr Ben Attig, Aymen Mabrouk, Tarek Cherni, Mounir Ben Moussa, Mohamed Sadok Boudaya

**Affiliations:** 1 Department of General Surgery, Jendouba Hospital, Jendouba, Tunisia; 2 Faculty of Medecine of Tunis, University of Tunis El Manar, Tunis, Tunisia; 3 Department of Surgery A, Thoracic Surgery, Charles Nicolle Hospital, Tunis, Tunisia; 4 Department of Surgery A, General Surgery, Charles Nicolle Hospital, Tunis, Tunisia

**Keywords:** echinococcosis hepatic, Echinococcus granulosus, hydatid cyst, thoracotomy, management

## Abstract

**Background:**

Liver dome hydatid cyst (LDC) presents a challenge for the surgeon and may be difficult to access through conventional laparotomy. Therefore, thoracic approach can be an effective alternative in these cases. This study aims to describe the postoperative outcomes of LDC operated via thoracotomy and to present the experience of one surgical center in Tunisia.

**Materials and methods:**

This is a retrospective study including all patients with a diagnosis of liver dome hydatid cyst and underwent surgery via a posterolateral thoracotomy, in the Department of Surgery “A” of the Charles Nicolle Hospital between 2018 and 2023.

**Results:**

Forty-nine patients were included in our study. The mean age was 52 years with a standard deviation of 19. The male to female ratio was 0.96. The main presenting symptoms were right upper quadrant abdominal pain, right chest pain and cough. A posterolateral thoracotomy was indicated in four scenarios: right lung and liver dome cyst, liver dome cyst with thoracic rupture, recurrent liver dome cyst previously underwent surgery via a laparotomy and the accessibility of the cyst. The morbidity rate was 12.2%. Follow-up showed one recurrence.

**Conclusion:**

Posterolateral thoracotomy appears to be a distinct and advantageous approach compared to traditional laparotomy, for the management of LDC, although the available evidence remains limited.

## Introduction

Liver hydatid cyst is a public health problem in endemic countries, such as Tunisia. This benign cystic lesion of the liver is caused by Echinococcus granulosus (1). However, it is considered a significant burden on the healthcare system due to serious complications, including cystobiliary communication, acute cholangitis, intraperitoneal rupture, and anaphylaxis. Surgery remains the standard of care for its management. Still, there is no clear consensus on the ideal surgical procedure. The choice of the surgical technique depends on the type and the presentation of the cyst (1, 2).

Liver dome cysts (LDC), located in the upper posterior segments, present a challenge for the surgeon and may be difficult to access through conventional laparotomy due to their anatomical position and the risk of thoracic migration, which can lead to pleural and pulmonary complications (3).

In light of these challenges, thoracotomy has been proposed as an alternative surgical approach. This technique offers the advantage of treating simultaneously the liver lesions and the thoracic complications or a pulmonary hydatid cyst, potentially eliminating the need for a second intervention or extensive abdominal surgery (4). However, it is essential to consider the consequences on both the abdominal and thoracic regions after surgery. The aim of this study is to describe the postoperative outcomes of liver dome cyst operated via thoracotomy and to present the experience of one surgical center in Tunisia.

## Materials and methods

A retrospective analysis was performed including all the patients admitted to the “A” Surgery unit of the Charles Nicolle Hospital in Tunis, Tunisia, between January 1, 2018 and December 31, 2023 with a diagnosis of liver dome hydatid cyst and underwent surgery via a posterolateral thoracotomy. The diagnosis was established based on clinical history, physical examination and imaging findings. One thoracic surgeon performed all the surgeries.

The study included patients over the age of 15, regardless of whether they had a pulmonary hydatid cyst. It also included patients undergoing surgery for the first time and those who had a recurrence after an initial abdominal surgery for a liver dome cyst. Patients who underwent thoracic surgery for liver lesions other than hydatid cyst were excluded.

The surgical approach was determined by a multidisciplinary team with expertise in both general and thoracic surgery.

A posterolateral thoracotomy was performed through whether the 5th, 6th, 7th or the 8th intercostals space. After entering the chest, the incision was extended internally along the intercostal space. Subsequently, a phrenotomy was performed to access the hepatic dome.

All information was collected from the records (observations, operative and radiological reports). Patient's clinical features, age, sex, comorbidities, laboratory and imaging findings and the management strategies were reviewed.

Statistical analyses were performed using the jamovi project (version 2.3). Continuous variables were tested for normality of distribution by means of Kolmogorov-smirnov test. They were expressed as mean and standard deviation when normally distributed, and as median and interquatril (IQI) when skewed.

This study was approved by the Ethics committee of Charles Nicolle Hospital of Tunis, Tunisia. The need for written informed consent was waived due to the retrospective nature of the study and the anonymity of the study population.

## Results

During the six-year period of our study, a total of 49 patients underwent surgery for liver dome hydatid cyst via a posterolateral thoracotomy. The demographic information and clinical presentation are summarized in Table 1. The mean age of study population was 52 ± 19.1 years, with a sex ratio of 0.96. Five patients had a history of prior laparotomy for LDC.

**Table 1:** Demographic and clinical characteristics of the study population (*n* = 49).

Variable	*n* (%) or mean ± SD
Age (years)	52 ± 19.1
Sex	
Male	24 (49%)
Female	25 (51%)
Comorbidities	
None	13 (26.5%)
Diabetes mellitus	5 (10.3%)
Hypertension	9 (18.4%)
History of previous laparotomy for liver dome hydatid cyst	5 (10.3%)
Presenting symptoms	
Right upper quadrant abdominal pain	12 (24.5%)
Chest pain	22 (44.9%)
Cough	15 (30.6%)
Fever	7 (14.3%)
Dyspnea	9 (18.4%)

SD, standard deviation.

The main presenting symptoms were right upper quadrant abdominal pain, right chest pain and cough. Most of the patients exhibited more than one symptom and only three patients were asymptomatic.

Chest x-ray was performed for all patients, abdominal ultrasound was performed in 16 cases and CT-scan was performed in all cases to evaluate the extent of the disease. CT imaging also allowed for accurate measurement of cyst size, with a mean diameter of 9.42 cm (range: 6–15 cm). In 11 cases, patients had separate liver and lung hydatid cyst. A diaphragmatic breach was identified in 11 patients. The results of imaging modalities are shown in Table 2.

**Table 2:** Radiological assessment of liver dome hydatid cysts showing the distribution of findings across different imaging modalities.

Modality	Findings (*n*)
Chest x-ray (49 patients)	Normal: 13
	Pulmonary opacity: 14
	Pleural opacity: 15
	Elevated diaphragm: 7
Ultrasound (16 patients)	**Liver cysts**
	Single cyst: 14 patients
	Multiple cysts: 2 patients (2 cysts each)
	**Gharbi Classification:**
	Type 1: 1
	Type 2: 2
	Type 3: 9
	Type 4: 6
	Type 5: 0
Ct-Scan (49 patients)	**Liver hydatid cyst**
	41 patients: 1 cyst
	6 patients: 2 cysts
	2 patients: 3 cysts
	**Liver and lung hydatid cyst:** 11 patients
	right lung: 11
	**complications:**
	thoracic rupture: 25
	diaphragmatic breach: 11
	biliary duct dilatation: 7

**Figure 1:**
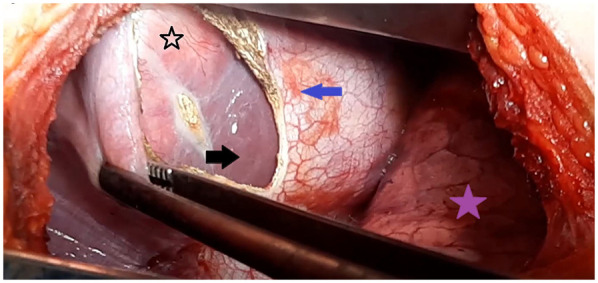
Intraoperative view showing the transthoracic approach for a liver dome hydatid cyst. The purple star indicates the right lung; the blue arrow marks the diaphragmatic incision (phrenotomy) providing access to the hepatic dome (black arrow); and the black star points to the protruding dome of the liver hydatid cyst.

The surgical approach was determined after clinical and imaging assessments. A posterolateral thoracotomy with phrenotomy (Figure 1) was selected in the following scenarios: right lung and liver dome cyst for 11 patients (23%), liver dome cyst with thoracic rupture in 25 cases (51%), recurrent liver dome cyst previously underwent surgery via a laparotomy in five cases (10%) and the accessibility of the cyst in eight cases (16%).

For the 11 patient who had separate liver and lung hydatid cyst, a cystectomy was performed for the pulmonary hydatid cyst. Cases involving liver dome cysts with thoracic rupture (51% of patients) were classified according to the Mestiri classification (Table 3). Among these, six patients were managed with pulmonary wedge resection, three patients with lobectomy, and another three patients with segmentectomy.

**Table 3:** Distribution of patients with thoracic migration of liver dome hydatid cysts according to the mestiri classification.

Type	Subtype	*n* (%)
Type I		5 (20%)
direct fistula of the cyst into the bronchi	IA: Small bronchial fistula	1 (4%)
	IB: Large bronchial fistula	4 (16%)
Type II		4 (16%)
intrapulmonary cavern	IIA: without bronchial fistula or bronchiolar fistula	0 (0%)
	IIB: with large-caliber bronchial fistula	4 (16%)
Type III		0 (0%)
intermediate intra-pleural collection	IIIA: No bronchial fistula	0 (0%)
	IIIB: With bronchial fistula	0 (0%)
	IIIC: fistulization towards the wall (empyema of necessity)	0 (0%)
Type IV		16 (64%)
Rupture into pleural cavity	IVA: Acute rupture: bilio-hydatid pleurisy	8 (32%)
	IVB: Secondary pleural hydatidosis	8 (32%)

For the liver dome cysts, surgical deroofing was the most common procedure, carried out in 41 patients (83.7%). A pericystectomy was performed in the remaining eight cases. A biliary fistula was identified in eight cases treated with simple suture.

Postoperatively, patients required subphrenic drainage for a mean duration of 4 days ±3.8. Thoracic drainage was maintained for a shorter period, averaging 2.6 days ±2.1. All patient were prescribed Albandazole at a dose of 400 mg twice daily for three cycles of 28 days, each separated by a 14-day interval.

In terms of post-operative complications, only three patients presented external biliary fistula that stopped on its own and other three patients developed a parietal infection. No reinterventions were necessary. Only one postoperative death occurred due to anaphylactic shock.

The mean follow-up duration was 3.7 months. Only one recurrence was observed within the study population. The patient was reoperated via an anterolateral thoracotomy.

## Discussion

Surgical management of hepatic hydatid cyst is a major challenge for the surgeon, requiring careful planning regarding the approach and the technique (5).

Surgery for a LDC is usually performed through an abdominal incision. However, the subdiaphragmatic region is difficult to access due to the presence of the rib cage. This specific anatomical feature limits exposure and compromises optimal control of the adjacent structures. Therefore, a phrenotomy performed via a posterolateral thoracotomy can be an effective alternative to provide direct access to the hepatic dome cyst (6). A thoracotomy is particularly indicated in cases of thoracic migration of the LDC because it allows for the simultaneous treatment of thoracic, diaphragmatic, and hepatic lesions (3, 5). Other indications for this procedure include the presence of hydatid cysts in both the liver and the lungs, recurrent hepatic hydatid cyst, and cysts that are accessible below the diaphragm (4, 7).

Symptoms such as right chest pain, cough, and right upper quadrant pain are the most frequently reported in the series of LDC with thoracic migration (8–10). However, some patients may remain asymptomatic.

Imaging modalities are necessary for evaluating patients with these symptoms. Chest x-rays are useful for diagnosing LDC by showing diaphragmatic elevation (4). Seven patients in our study had changes in the appearance of the diaphragm. Ultrasound remains valuable in the case of hepatic hydatid cyst and in identifying complications. It is the method of choice due to its accessibility, however, it depends on physician's technique and experience (11).

Yet, a CT scan has demonstrated a 94% sensitivity rate (12). It plays a crucial role in the perioperative period especially for mapping hepatic hydatid lesions and assessing surrounding structures (13). CT scans can detect diaphragmatic breachs, which suggest migration of the cyst into the chest. This was observed in 11 patients in the present study. However, surgery revealed 22 diaphragmatic breachs. This underestimation highlights a limitation of CT in identifying diaphragmatic defects, especially in case of chronic inflammatory changes. Some authors reported that CT scan may underestimate thoracic migration when the cyst is adherent or when diaphragmatic continuity is not completely interrupted (13). Although CT provides excellent anatomical definition, small transdiaphragmatic communications or thoracic extensions can be missed, particularly in chronic or partially ruptured cysts (12, 14).

There are no established guidelines for treating liver hydatid cysts. Currently, laparotomy is the standard approach (15). However, thoracotomy provides better exposure for liver dome cysts. A phrenotomy allows direct access to liver lesions and is particularly effective for recurrent cysts after abdominal surgery (16).

Studies show that a thoracic approach is effective for patients with both lung hydatid cysts and liver dome cysts, enabling them to avoid a second surgery (4, 7, 17).

Posterolateral thoracotomy allows the surgeon to conduct a thorough assessment of pleural, pulmonary, diaphragmatic, and subphrenic lesions (18). In our series, the posterolateral thoracotomy was used for all patients, passing through the lower intercostal spaces (7th or 8th) in 87.7% of cases.

The second surgical approach reported in the literature was anterolateral thoracotomy. Lone et al. previously described this technique in a study involving 25 patients who underwent surgery for a concomitant right lung hydatid cyst and liver dome cyst (19). Although anterolateral thoracotomy is a viable option for certain thoracic procedures, it has limited visibility.

Other authors have examined the role of minimally invasive surgery as well. In 2013, Kumbhar et al. (20) proposed a combination of laparoscopic and thoracoscopic approaches for the treatment of liver hydatid cysts, reporting favorable outcomes with this technique. Similarly, Kanojia and Bawa (21) described two cases in which double localization in the right lung and liver was treated using a combination of thoracoscopy and a phrenotomy, with no recurrence observed. However, it is important to take careful precautions when using minimally invasive techniques to prevent contamination of the surgical field by fertile hydatid material. These precautions are important to prevent recurrence and the formation of fluid collections, which can complicate postoperative recovery and outcomes (22).

In our series, 11 patients presented with lung hydatid cysts and liver dome cysts without thoracic migration. For these patients, a thoracic approach was recommended to avoid the need for two separate interventions. This method facilitated quicker recovery (23, 24). Our approach yielded favorable results, aligning with the findings of Kurul et al. Among their 405 patients with a right lung and liver hydatid cysts, only 12 patients (3%) experienced complications, including biliary and bronchial fistulas (25).

Thoracic lesions secondary to liver dome cysts occur when there is thoracic migration through the diaphragm (26). In our study, these lesions were more frequently observed in the pleura and ranged from pleural symphysis to secondary pleural hydatid cyst.

The variation in thoracic lesions has led to different classifications: Mestiri's classification in 1987 (10), and Gomez's classification in 1995 (27). In the present study, we adopted the Mestiri classification. Notably, it was found that 64% of patients with thoracic migration of LDC were classified as type IV. Regarding Gomez's classification, it may lack precision, but it has the advantage of considering some diaphragmatic lesions (27).

The presence of a LDC does not necessarily indicate a diaphragmatic lesion. Several factors influence this, including the cyst's size, the inflammatory response it generates, the presence of infection, any potential biliary fistulas that may cause chemical irritation, and the duration of contact between the cyst and the diaphragm (6). The main observed lesion is a diaphragmatic breach, which was present in 44.9% of our patients.

The surgical technique for treating LDC via thoracotomy is similar to the abdominal approach. It depends on the condition of the cyst and any liver lesions caused by the parasite. The most common procedure performed is cystectomy with resection of the protruding dome, as reported by Kabiri et al. (78.9%) (28), which aligns with our findings (83.7%).

According to Mestiri's classification, type I and II can cause varying degrees of lung damage. In our study, pulmonary resection was necessary for 12 patients (three lobectomies, three segmentectomies, and six wedge resection), accounting for 24% of cases with thoracic migration of LDC. These rates were 37% in a Tunisian study (9) and 26% in a large Moroccan study of 123 patients (28).

LDC surgery via thoracotomy can lead to many complications. These complications may be specific to liver injury (such as bile leak and phrenic collection), related to intra-thoracic lesions due to migration of LDC (including ventilation disorders and pneumothorax), or non-specific (such as wall infection, postoperative bleeding, and pulmonary embolism). In our study, the overall morbidity rate was 12.2%. In comparison, the morbidity rates reported in Şahin et al. series were 4.1% (7), and in the Kabiri et al. series, it was 14.6% (28).

In LHC surgery, biliary fistula is a significant complication, manifesting in 50% to 63% of cases (29). After transthoracic surgery for LDC, this complication is exacerbated by the negative pressure of the pleural cavity. Biliary fistula can result in prolonged hospitalization and may be challenging to manage. To prevent bile leaks, it is imperative to conduct a thorough examination of the pericyst and liver sections. Despite rigorous preventive measures during surgery, the occurrence of bile leaks within a few hours or days following the procedure remains a possibility (29). The drainage of the biliary leak can be facilitated by endoscopic sphincterotomy performed under endoscopic retrograde cholangiopancreatography. The objective of this procedure is to reverse the flow of bile into the bile ducts, depending on the location of the fistula. In the series by Saritas et al. (30), the rate of secondary bile fistula depletion after LDC surgery was 81%, with an average duration of 17.8 days. In the present study, postoperative bile leakage occurred in three patients (6.1%). None of them needed endoscopic treatment. This low incidence may be explained by meticulous inspection of the residual cavity, direct suture of bile duct openings, and systematic placement of subphrenic drainage to prevent bile accumulation. In addition, maintaining chest drainage for a short period after surgery helped equalize the pressure. Our results are consistent with previous studies reporting biliary fistula rates ranging from 4% to 15% following thoracic or combined approaches (7, 23, 29).

Our study has several limitations. First, it was a retrospective study. Therefore, data might be missing and it was difficult to accurately report subjective symptoms and signs. However, the fact that all patients in the series were managed by one single surgeon partially limited the impact of this bias. Second, it was a single-center study with a small number of patients. But this is one of the few Tunisian studies that has focused on this subject and has allowed for the collection of a significant number of patients over a relatively short period. Third, the absence of a control group of patients who underwent abdominal surgery constitutes another limitation of this study. However, our objective was not to establish superiority, but rather to report our institutional experience in carefully selected cases where the transthoracic approach was considered more appropriate due to cyst location, recurrence after previous abdominal surgery, or associated thoracic involvement. Also, one of the main limitations of our study is the relatively short follow-up period, with a mean duration of 3.7 months. Although hydatid disease is known to potentially recur several years after treatment, making long-term surveillance essential, the primary objective of this study was to evaluate the immediate safety and feasibility of the thoracic approach in LDC. Further research, including multicenter studies, is needed to determine the best therapeutic approach in case of LDC.

## Conclusion

The current study contributes significantly to our understanding of thoracotomy for liver dome hydatid cysts. Thoracotomy appears to be a feasible approach, particularly for complex or recurrent cysts. Therefore, changing preoperative protocols that includes cross-sectional imaging for all suspected dome cysts could improve surgical outcomes.

The management of LDC remains challenging. Posterolateral thoracotomy has demonstrated potential benefits in patients with thoracic migration, previous abdominal surgery, or concomitant pulmonary hydatid disease. However, further studies are needed to provide conclusive evidence and establish clear guidelines for the management of LDC.

## Data Availability

The raw data supporting the conclusions of this article will be made available by the authors, without undue reservation.
